# Epoxy-Acrylic Polymer In-Situ Filling Cell Lumen and Bonding Cell Wall for Wood Reinforcement and Stabilization

**DOI:** 10.3390/polym16010152

**Published:** 2024-01-03

**Authors:** Yiliang Liu, Jilong Fan, Fengbiao Yao, Xudong Gao, Yueying Zhao, Baoxuan Liu, Xiaoying Dong, Yongfeng Li

**Affiliations:** 1 Key Laboratory of State Forestry Administration for Silviculture of the Lower Yellow River, College of Forestry, Shandong Agricultural University, Tai’an 271018, China; liuyiliang0207@163.com (Y.L.); sdfjl1235@126.com (J.F.); fengbiaoyao@163.com (F.Y.);; 2 Shandong Everjoy Technology Material Co., Ltd., Jining 277600, China; xgsyyf@126.com; 3 Shandong Laucork Development Co., Ltd., Jining 272100, China; liubaoxuan123@126.com; 4 Key Laboratory of Bio-Based Material Science and Technology of Ministry of Education, Northeast Forestry University, No. 26 Hexing Road, Harbin 150040, China

**Keywords:** wood, formaldehyde-free resin, composite, interface strengthening, in situ filling

## Abstract

Under a global carbon-neutralizing environment, renewable wood is a viable alternative to non-renewable resources due to its abundance and high specific strength. However, fast-growing wood is hard to be applied extensively due to low mechanical strength and poor dimensional stability and durability. In this study, epoxy-acrylic resin-modified wood was prepared by forming a functional monomer system with three monomers [glycidyl methacrylate (GMA), maleic anhydride (MAN), and polyethylene glycol-200-dimethylacrylic acid (PEGDMA)] and filling into the wood cell cavity. The results showed that in the case of an optimal monomer system of nGMA:nPEGDMA = 20:1 and an optimal MAN dosage of 6%, the conversion rate of monomers reached 98.01%, the cell cavity was evenly filled by the polymer, with the cell wall chemically bonded. Thus, a bonding strength of as high as 1.13 MPa, a bending strength of 112.6 MPa and an impact toughness of 74.85 KJ/m^2^ were applied to the modified wood, which presented excellent dimensional stability (720 h water absorption: 26%, and volume expansion ratio: 5.04%) and rot resistance (loss rates from white rot and brown rot: 3.05% and 0.67%). Additionally, polymer-modified wood also exhibited excellent wear resistance and heat stability. This study reports a novel approach for building new environmentally friendly wood with high strength and toughness and good structural stability and durability.

## 1. Introduction

The application of green and renewable resources is of great significance to sustainable development and the ecological environment [1,2,3,4]. Wood, one of the most abundant biomass resources, is renewable and degradable, which plays an important role in achieving carbon neutrality [5,6]. Aspen wood, eucalyptus wood and other fast-growing wood species present wide sources and sufficient reserves, which are worthy of full utilization, high-value development and extensive application. However, such wood species usually have a low mechanical strength and poor structural stability and rot resistance. Therefore, these wood were normally used as low-end raw materials such as firewood and non-load-bearing furniture plates, which restricts the realization of their full potential [7].

The resin impregnation of wood cell cavity/wall is a common solution for enhancing mechanical properties, dimensional stability, rot resistance and durability of fast-growing wood. For example, vinyl monomers, such as methyl methacrylate and styrene from wood plastic composites in wood cell cavities, significantly improved the above synthetical properties of wood [8,9,10]. Such monomer polymerization is based on free radical polymerization, which presents the advantage of fast reaction speed, no solvents and no residual by-products. However, the improvement in dimensional stability and durability of wood plastic composites is limited and uneconomical. Such wood plastic composites show poor conversion rate, high cost and poor interfacial compatibility due to their volatility and low polarity, which restrict the practical application of vinyl monomer modification. Meanwhile, another corresponding widely explored resin system is the aqueous aldehyde oligomers system, including phenol-formaldehyde (PF) resin, urea-formaldehyde resin and melamine-formaldehyde resin [11,12,13,14,15]. These oligomers formed crosslinked solid polymer networks through condensation polymerization with water as the carrier. This system presents advantages such as low cost, controllable process, good interfacial compatibility between polymers and wood, good synthetical properties and high-cost performance [12,16,17]. However, the release of formaldehyde from modified wood restricts its extensive application. Moreover, recently reported research on resins (both vinyl monomers and aqueous aldehyde oligomers) modified wood present high brittleness, which leads to low impact toughness, making it difficult to be used as structural material and realize its high-value application [18,19,20].

Regarding this, in this study, an organic unsaturated monomer system with low volatility was designed based on the principle of free radical polymerization to construct high-performance modified wood with good interfacial compatibility between polymer and wood, high impact toughness, no formaldehyde release and compatible mechanical strength, dimensional stability and durability. Via free radical polymerization, maleic anhydride (MAN) acts as an interface modifier and a catalyst, glycidyl methacrylate (GMA) acts as an interface modifier and a rigid segment source monomer and polyethylene glycol-200-dimethylacrylic acid (PEGDMA) acting as a flexible segment source monomer, the conversion rate in wood is as high as 98.03%. Moreover, the mechanical strength, impact toughness, dimensional stability, rot resistance and heat stability of modified wood were significantly improved compared with original wood, indicating that composites prepared by the optimal system presented better synthetical properties. This result was similar to our previous work but achieved superior results [21]. These results indicated that the technical strategy reported in this study is hopeful to realize the high-value application of low-quality wood in the field of structural wood.

## 2. Materials and Methods

### 2.1. Materials

Populus ussuriensis Komarov with a size of 25 mm × 300 mm × 2000 mm [radial (R) × tangential (T) × longitudinal (L)] and a mean density of 0.33 g/cm^3^ was purchased from Maoershan Experimental Forest Farm, Heilongjiang, China, was used as aspen wood samples. The rotary-cut veneer of aspen wood (size: 300 mm × 300 mm × 3 mm, mean density: 0.35 g/cm^3^) was purchased from the wood market in Tai’an, China. Medium-density fiberboards (size: 300 mm × 300 mm × 18 mm, density: 0.68 g/cm^3^), shaving boards (size: 300 mm × 300 mm × 16 mm, density: 0.72 g/cm^3^) and plywood (size: 300 mm × 300 mm × 9 mm) were purchased from Nancha Wood Based Panel Factory in Tai’an, China.

GMA (molecular weight value is 142.15), azodiisobutyronitrile (AIBN, molecular weight value is 164.208), MAN (molecular weight value is 98.057) and PEGDMA (molecular weight value is 1079.27) were bought from Shanghai Yuanji Chemical Co., Ltd., Shanghai, China, Tianjin Kemiou Chemical Reagent Development Center, and Yantai Yunkai Chemical Co., Ltd., Yantai China, respectively. Acetone and tetrahydrofuran were purchased from Tianjin Kaitong Chemical Reagent Co., Ltd., Tianjin, China. The above reagents were analytical grade and directly used without further purification.

### 2.2. Method

#### 2.2.1. Polymer and Composite Preparation

An unsaturated monomer system was prepared by the following method: The GMA and PEGDMA monomers were mixed at a molar ratio of 2:1 and stirred evenly. Then, AIBN, accounting for 1% of the total mass of the two monomers, was added and stirred until dissolved to form a colorless and transparent solution.

The functional monomer system was prepared by dissolving MAN acetone at different concentrations of 2%, 4%, 6%, 8% and 10%, and the resultant solution was added to the preferred unsaturated monomer system described above and uniformly stirred to form a colorless transparent solution.

Afterward, polymer-modified veneered man-made boards were prepared. The poplar wood veneer was immersed in the prementioned solution and treated under vacuum at 0.08 MPa for 20 min, then under nitrogen atmosphere at 0.8 MPa for 20 min, and then the veneers were hot pressed at 130 °C, 0.8 MPa for 15 min. This was designed for polymerization of monomers in a veneer of aspen wood and bonding between the veneer and the substrate. The interface bonding performance between the veneer and the substrate was judged based on the bonding strength.

Finally, polymer-modified wood was prepared as follows: The aspen wood was separately immersed in the above monomer systems. Subsequently, the polymer-modified wood was treated under a vacuum of 0.08 MPa for 20 min, then under a nitrogen atmosphere of 0.8 MPa for 20 min, and then heated at 80 °C for 8 h and 110 °C for 8 h, in turn, to prepare into polymer-modified (through in situ filling) aspen wood, which was labeled as Wood-P (GMA-co-PEGDMA) composites and Wood-P (MAN-GMA-co-PEGDMA) composites, respectively.

#### 2.2.2. Physical and Mechanical Properties Testing

A universal testing machine (AG-10TA, Shimadzu, Kyoto, Japan) was employed to test the mechanical properties and surface bonding strength of various wood materials. The test methods were conducted according to the GB/T 17657-2013. The size of the specimen used for bending strength measurement is 20 mm × 20 mm × 300 mm (R × T × L); the size of the specimen used for compressive strength test is 20 mm × 20 mm × 30 mm (R × T × L); the size of the specimen used for impact toughness test is 20 mm × 20 mm × 300 mm (R × T × L); The size of the sample used to test the bond strength is 50 mm × 50 mm × 20 mm (R × T × L). Each test was performed 5 times in parallel, and the average value was taken. The mechanical properties of untreated and treated samples were tested using a universal testing machine (AG-10TA, Shimadzu). For testing of gluing strength, bending strength and compressive strength, a uniform load of 1 mm/min was applied until the specimen was completely destroyed. For the hardness test, an indenter (11.3 mm diameter steel ball) attached to the loading plate of the tester was pressed against the surface of the specimen. A preload of 1~2 N was applied to stabilize the specimen. Then, the load was increased to reach a target of 1000 N within 15 s and held for 25 s. The actual contact area under the indentation was used to calculate the hardness of the specimen. Load-deflection data were obtained at a sampling rate of 10 data points per second. The pendulum was placed at an angle of 150°, and the energy was set to 5 J so that the pendulum fell freely to destroy the specimen to test impact toughness. Dimensional stabilization: The specimen size was 20 mm × 20 mm × 20 mm (R × T × L). The samples were immersed in distilled water for 720 h. The sample was then removed to measure the dimensions of the sample and the change in weight.

#### 2.2.3. Wood Preservation Testing

The wood preservation test was carried out according to the People’s Republic of China (PRC) Forestry Industry Standard-Method of Laboratory Test for Toxicity of Wood Preservatives to Soft-rot Fungus (LY/T1283-1998). The brown rot fungus was *Gloeophyllum trabeum* (Pers. ex fr.) Murr., and the white one was *Coriolus versicolor* (L.:Fr.) Quél. The size of the test pieces was 20 mm × 20 mm × 10 mm (R × T × L). The duration of the preservation test was 12 weeks. Each wood material was tested 5 times in parallel, and the average value was taken.

#### 2.2.4. Morphology and Structure

The microstructure of test pieces was characterized by a scanning electron microscope (SEM, QUANTA2000, FEI, Hillsboro, NH, USA). Additionally, the functional groups, crystallinity and thermal decomposition ability of test pieces were tested by attenuated total reflection-Fourier Transform Infrared Spectroscopy (ATR-FTIR) (MagnaIR560, Nicolet, MN, USA), X-ray diffraction (XRD) (D/max2200, Rigaku, Tokyo, Japan) and thermogravimetry (TG)/derivative thermogravimetry (DTG) (Pyris 6 TGA, Perkin Elmer, Waltham, MA, USA), respectively.

The micromorphology of the wood was observed using a scanning electron microscope (SEM, QUANTA2000, FEI, Hillsboro, NH, USA). Prior to analysis, the samples were gold-sprayed with a 10 mA current for 15 s. The chemical composition of the samples was detected by Fourier Transform Infrared Spectroscopy (FTIR, Magna IR560, Nicolet, Madison, WI, USA) with a resolution of 4 cm and a total of 40 spectra was accumulated. The crystallinity of the samples was determined by X-ray diffraction (XRD, D/MAX2200VPC, Rigaku, Tokyo, Japan). XRD tests were performed at 40 kV and 30 mA. Figures were collected in the 2θ range from 5–60° with a scanning speed of 5 °C min^−1^. The thermal stability of the specimens was determined using a thermogravimetric analyzer (TGA, Q500, Waters, Taunton, MA, USA). The specimens were heated from 35 °C to 600 °C at a heating rate of 10 °C min^−1^ under a stream of nitrogen (100 mL min^−1^).

## 3. Results and Discussion

**Figure 1:**
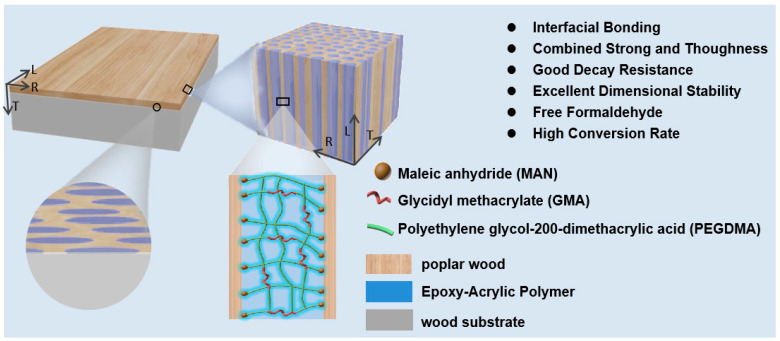
Schematic diagram of epoxy-acrylic polymer-modified (through in situ filling) aspen wood realizing compatibility of interface bonding and high performance.

In order to test the interfacial compatibility between polymer and wood, an epoxy-acrylic polymer was designed to realize interface bonding between the veneer and the substrate. The epoxy-acrylic polymer-filling veneer and gluing of the man-made board substrate to form modified veneered man-made board composites are shown in Figure 1. In this study, formaldehyde-free GMA, PEGDMA and MAN monomers were selected to prepare a functional monomer system. With epoxy groups, GMA molecules played the role of “interface bonding”. With a long flexible segment, PEGDMA could enhance the brittleness of polymer and the impact toughness of wood. MAN served as a crosslinking accelerator to improve the ring-opening bonding ability of GMA and enhance the interfacial compatibility between polymer and wood substrate [21,22].

### 3.1. Optimization of Functional Monomer Ratio

#### 3.1.1. Optimization of GMA/PEGDMA Ratio

**Figure 2:**
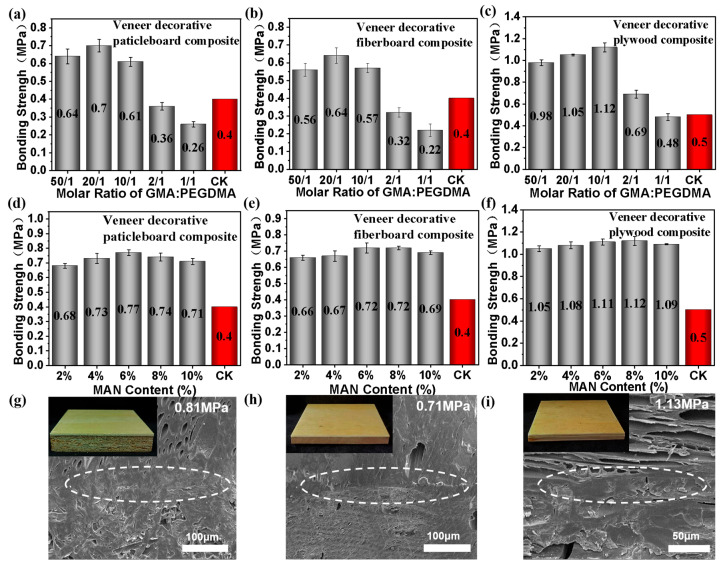
Optimization of monomer ratio based on the interfacial bonding strength between three kinds of man-made board substrates and modified veneer: optimization of GMA/PEGDMA ratio based on (**a**) paticleboard, (**b**) fiberboard and (**c**) plywood; optimization of MAN dosage based on (**d**) paticleboard, (**e**) fiberboard and (**f**) plywood; microstructure of the bonding interface of modified veneered (**g**) paticleboard, (**h**) fiberboard and (**i**) plywood at the optimal ratio.

The bonding strength is the major index for measuring the interface bonding performance, and a higher bonding strength suggests better interface bonding between two phases. In this study, GMA-PEGDMA monomer system-modified veneered man-made board composites (Figure 2a–c) were prepared first. For composites with shaving boards and medium-density fiberboards as the substrate, the bonding strength is less than that (0.40 MPa) specified in national standards when nGMA:nPEGDMA ≤ 2:1, whereas it is higher than that in national standards when nGMA:nPEGDMA ≥ 10:1 (Figure 2a,b). Overall, the bonding strength of the modified veneered man-made board composites reached the peak, which was 0.70 MPa and 0.64 MPa, respectively, when nGMA:nPEGDMA = 20:1. Additionally, for modified veneered man-made board composites with plywood as the substrate, the bonding strength declined with the decrease in monomer ratio when nGMA:nPEGDMA ≤ 10:1, which reached the lowest value (0.48 MPa), slightly smaller than that in national standards (0.50 MPa), when nGMA:nPEGDMA = 1:1, and peaked (1.12 MPa) when nGMA:nPEGDMA = 10:1 (Figure 2c). Generally, bonding strength depends on the cohesion of the polymer itself and its interfacial bonding ability with the substrate. Higher GMA content indicated a greater probability of polymer bonding to wood and better interfacial bonding. The higher the content of PEGDMA was, the more fully crosslinked the polymer itself, and the stronger the cohesion would be, which was also conducive to bonding performance. Only when the contribution of the reaction system to ‘cementation’ and ‘crosslinking’ is balanced can the bonding strength peak. Obviously, under the same process conditions, for composites with shaving boards and medium-density fiberboards as the substrate, the bonding strength peaked when nGMA:nPEGDMA = 20:1, which far exceeds that in national standards. When plywood was used as the substrate, the bonding strength peaked when nGMA:nPEGDMA = 10:1, but the bonding strength at nGMA:nPEGDMA = 20:1 still far exceeds that in national standards. Therefore, the optimal ratio between functional monomers was finally determined as nGMA:nPEGDMA = 20:1 for the sake of research unity.

#### 3.1.2. Optimization of MAN Usage

Furthermore, the optimal dosage of MAN was determined based on the bonding strength at the optimal GMA-PEGDMA monomer ratio. The results are shown in Figure 2d,e, which reveal that when MAN content increases from 2% to 10% (weight percent of the total mass of the two functional monomers), the bonding strength of composites with shaving boards and medium-density fiberboards as the substrate rises and then declines; however, it is higher than that of boards without MAN (Figure 2a,b), proving that the MAN presents a positive contribution to improve bonding strength. From the view of molecular structure, MAN is an a,b-unsaturated carboxylated compound containing one carbon-carbon double bond (C=C) and two carboxyl groups (ACOOA). This highly reactive conjugated structure enhances the reactivity of MAN with wood and polymer matrices, resulting in crosslinking or strong adhesion at the interface. Theoretically, MAN has a reactive anhydride group that can react with hydroxyl groups in the wood cell wall through nucleophilic substitution to form new carboxyl grafts in the wood cell wall. The newly formed carboxyl group can be used not only as a nucleophilic reagent but also as a catalyst to provide a certain acidity to promote the nucleophilic substitution reaction between GMA and carboxyl and even hydroxyl groups in the wood cell wall. Therefore, MAN facilitated the grafting, polymerization and crosslinking of functional monomers in wood, further reflected in the improvement of bonding strength. However, when the MAN dosage exceeds a certain limit, the density of the polymer crosslinking point will be too high, with increased brittleness and decreased grafting probability, resulting in decreased bonding strength. Hence, under the MAN content of 6%, the crosslinking and grafting of the functional monomer system achieved a balance, and the bonding strength reached the maximum, which was 0.77 MPa and 0.72 MPa, respectively. In addition, a similar rule was also found in the results of composites with plywood as the substrate (Figure 2f). In other words, all in all, the addition of MAN further improved the bonding strength of composites. The bonding strength rose with the increase of MAN at the dosage of less than 8% and then dropped when the dosage exceeded 8%. The bonding strength peaked (1.12 MPa) at the dosage of 8%, showing little difference from that at 6%.

Overall, the bonding strength of Wood-P (MAN-GMA-co-PEGDMA) composites was improved by adding MAN. Composites with plywood as the substrate displayed the highest surface bonding strength, followed by those with shaving board as the substrate, while composites with medium-density fiberboard as the substrate had the lowest surface bonding strength. This rule is also consistent with the above test results. Considering the cost, the optimal dosage of MAN was finally determined to be 6%.

Through the above single-factor experiments, it was determined that the optimal ratio of the functional monomer system was nGMA: nPEGDMA = 20:1, and the optimal dosage of MAN was 6%. According to Figure 2g–i, under the optimal conditions, the ‘bonding’ interface of resin-modified veneered man-made board composites with the three kinds of man-made boards as the substrate is well-bonded without obvious interface gaps, suggesting that the interface interaction force is strong and the superior bonding is achieved through the chemical reaction of the functional monomer system, which is in line with the actual bonding strength results.

### 3.2. Interface Bonding of Modified Wood and Its Influence

#### 3.2.1. Comparison of Resin Conversion Rate

**Figure 3:**
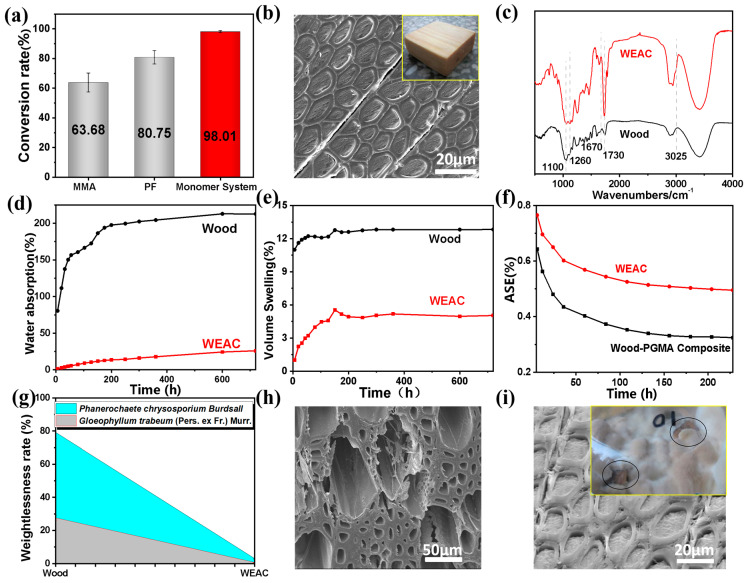
Dimensional stability and rot resistance of epoxy-acrylic polymer-modified wood: (**a**) comparison of resin conversion rate between monomer system and wood impregnated in other resins, (**b**) SEM microstructure of the cross-section of the modified wood, (**c**) FTIR curves of modified wood and unmodified wood, (**d**) water absorption rate, (**e**) water absorption volume expansion rate and (**f**) anti-expansion rate, (**g**) rot resistance value of modified wood and unmodified wood, (**h**) SEM microstructure of unmodified wood after wood preservation test, (**i**) SEM microstructure of modified wood after wood preservation test (the inner illustration is the image of modified wood not corroded by rot fungi in the wood preservation test).

After determining the optimal ratio of the functional monomer system, epoxy-acrylic polymer-modified wood was prepared. As shown in Figure 3a, the conversion rate of the functional monomer system in wood cell cavities is as high as 98.01% (Equation (1)), which is much higher than that of MMA monomer (63.68%) and PF resin (80.75%). In the functional monomer system, monomers present weak volatility, and there are many polymerization crosslinking sites. As a result, the resin volatility was lower (1.99%) during curing, and the resin was basically completely cured. However, free phenol and formaldehyde in PF resin are volatile, so 19.25% of the resin is volatilized during curing. MMA monomer has a low boiling point, and strong volatility causes 36.32% of it to be unconverted during curing. These results fully signify that the functional monomer system effectively solves the bottleneck problems of monomer volatility and low conversion rate.

RCR = m_2_/m_1_ × 100% (1)

In the equation: RCR—Resin Conversion rate.

m_1_—the weight of the monomer system.

m_2_—the weight of polymer converted from the monomer.

#### 3.2.2. SEM Characterization

It can be clearly observed from Figure 3b that the cell cavity of Wood-P (Man-GMA-co-PEGDMA) composites is evenly filled with the polymer, demonstrating sufficient and reasonable impregnation and thermal catalysis. In addition, the polymer is in close contact with the wood cell wall, suggesting that there may be a strong interfacial interaction between the two [23,24].

#### 3.2.3. FTIR Analysis

It was found that in contrast with Wood, Wood-P (Man-GMA-co-PEGDMA) composites exhibited an evidently enhanced carbonyl stretching vibration absorbance at the wave number of 1730 cm^−1^, the broadened C-O stretching vibration peaks at 1100 cm^−1^ and 1260 cm^−1^ (Figure 3c), indicating that monomers interact with wood during polymerization, thus affecting the vibration of original characteristic functional groups in wood components. It was inferred, combined with the reaction principle, that the epoxy group in the optimal functional monomer system can react with the hydroxyl group of wood components. When the optimal functional monomer system participates in monomer polymerization through its own double bond, the formed polymer may be grafted on the wood cell wall, thereby increasing the ratio of carbonyl and C-O bonds on the substrate of composites compared with the two functional groups of original wood components. Hence, the stretching vibration peaks of the two functional groups on the spectra were significantly enhanced. In contrast with Wood, Wood-P (MAN-GMA-co-PEGDMA) composites had a stronger carbonyl peak (compared with the C-H stretching vibration peak on their own -CH_3_), a slightly enhanced C(=O)–O stretching vibration peak at 1260 cm^−1^, a weak =C–H stretching vibration peak at 3025 cm^−1^ and a new weak peak at 1670 cm^−1^, suggesting that MAN participates in the chemical reaction of functional monomers, and makes positive contribution to the grafting polymerization of monomer system in wood. Compared with Wood, epoxy-acrylic resin-modified wood displayed broader ether bond stretching vibration peaks at 1100–1260 cm^−1^, which is closely related to the contribution of PEGDMA to the ether bond functional group of the system.

Based on the results of the above FTIR analysis and the reaction principle, it can be concluded that the functional monomer system composed of MAN, GMA and PEGDMA is polymerized in wood and involved in the grafting reaction of wood components, resulting in strong chemical bonding between the polymer and the wood cell wall, which is in line with the conclusion obtained by SEM observation.

#### 3.2.4. Dimensional Stabilization

Furthermore, the dimensional stability of epoxy-acrylic resin-modified wood was tested and presents a much lower water absorption rate than that of unmodified wood within 228 h after soaking in water. The water absorption rate of Wood-P (Man-GMA-co-PEGDMA) composites was 26% at 720 h after soaking in water, only 12.50% of that of wood (Figure 3d). Additionally, the water absorption volume expansion rate of Wood-P (Man-GMA-co-PEGDMA) composites was much lower than that of Wood, reaching 5.04% at 720 h after soaking in water, only 64.00% of that of Wood (Figure 3e). The anti-expansion rate of Wood-P (Man-GMA-co-PEGDMA) composites and Wood both decreased with time during soaking, which is different from the water absorption rate and the water absorption volume expansion rate. However, the anti-expansion rate of Wood-P (Man-GMA-co-PEGDMA) composites was always higher than that of Wood, which was 32.4% at 228 h, about 1.44% of that of wood. These results signify that Wood-P (Man-GMA-co-PEGDMA) composites have far superior water resistance to Wood. The wood cell cavity was filled, and the wood cell wall components were coated and grafted with the optimal functional monomer system through grafting polymerization, blocking the passage of water infiltration into the wood cell wall and improving the hydrophobicity of the substrate to a great extent. As a result, the dimensional stability of modified wood was obviously improved [21].

#### 3.2.5. Anti-Corrosion Properties

As shown in Figure 3g, the rot resistance of Wood-P (Man-GMA-co-PEGDMA) composites to brown and white rot fungi is increased by 96.15% and 97.57%, respectively, compared with that of Wood. As shown in Figure 3h,i, the substrate of Wood seriously collapsed after rotting by brown rot fungi, with visible hyphae on the cell wall. However, Wood-P (Man-GMA-co-PEGDMA) composites still presented a complete microstructure without obvious signs of rotting on the cell wall, which is consistent with the results of the mass loss rate. The preservation test images of Wood-P (Man-GMA-co-PEGDMA) composites also show that only a few brown rot fungi grew on the wood surface. It is because the epoxy-acrylic polymer makes the water content in the cell wall lower than that required by fungi for normal survival and blocks the path of fungi eroding the wood cell wall at the same time. Hence, Wood-P (Man-GMA-co-PEGDMA) composites exhibited significantly higher rot resistance than unmodified wood [22].

In short, the functional monomer system with a high conversion rate improved the interfacial compatibility between the polymer and the wood cell wall by polymerization after entering the wood cell cavity, thus significantly enhancing the dimensional stability and rot resistance of modified wood.

### 3.3. Impact Toughness and XRD Analysis of Modified Materials

#### 3.3.1. XRD Analysis

**Figure 4:**
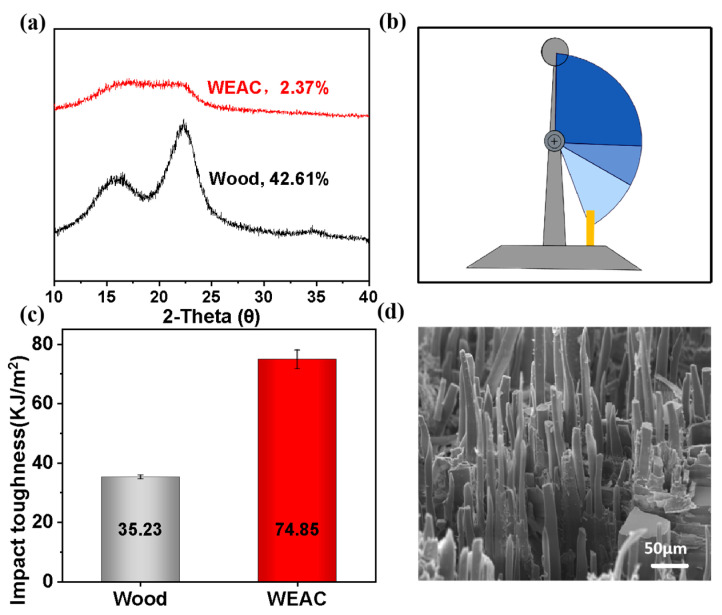
Impact toughness of polymer-modified wood: (**a**) XRD curves of polymer-modified wood and unmodified wood, (**b**) schematic diagram of impact toughness test, (**c**) comparison of impact toughness between polymer-modified wood and unmodified wood and (**d**) SEM microstructure of impact fracture surface of polymer-modified wood.

According to Figure 4a, both polymer-modified wood and unmodified wood show diffraction peaks representing the (101) and (002) crystal planes of cellulose near 2θ = 16° and 22°, implying that the polymer does not change the crystal structure of wood. However, compared with those of Wood, the diffraction peaks representing the (101) and (002) crystal planes of Wood-P (Man-GMA-co-PEGDMA) composites almost overlap with each other, suggesting that the relative crystallinity of cellulose in Wood-P (Man-GMA-co-PEGDMA) composites is lower than that of Wood. The relative crystallinity of cellulose of the two materials was calculated according to the Segal method (Equation (2)), and the results uncovered that the relative crystallinity of unmodified and modified wood is 42.61% and 2.37%, respectively, which is consistent with the difference in 2θ diffraction curve.

From the point of view of molecular structure, theoretically, the polymer formed by monomers is mostly amorphous due to their free radical polymerization in wood. After entering the cell, the polymer only grafted with the cell wall without changing the original structure of wood, resulting in more amorphous compounds in cell wall components. Hence, the relative content of cellulose dropped, reflected by the decrease in relative crystallinity. The higher the content of graft polymer is, the lower the relative crystallinity will be [24]. Therefore, the relative crystallinity of polymer-modified wood dropped.

C_r_I = (I_002_ − I_am_)/I_002_ × 100% (2)

In the equation—C_r_I is the percentage of relative crystallinity.

I_002_—(002) lattice diffraction angle of the maximum intensity (any unit).

I_am_ represents the scattering intensity of the amorphous background when the 2θ angle is close to 18 °, which is the same as the I_002_ unit.

#### 3.3.2. Impact Toughness

The impact toughness of polymer-modified wood was tested, and the results reveal that the impact toughness of Wood-P (Man-GMA-co-PEGDMA) composites is 2.12 times that of unmodified wood (Figure 4c), indicating that the polymer acts as a reinforcement and significantly improves the impact toughness of wood. Additionally, the SEM microstructure of the fracture surface in Figure 4d also shows that Wood-P (Man-GMA-co-PEGDMA) composites have obvious ‘shrinkage’ fracture traces under impact stress, indirectly suggesting good impact toughness of the material. Therefore, it was considered that under the optimal system, the polymer structure and interface worked together to improve the impact toughness of wood. The former was suggested to be the main influencing factor [23].

### 3.4. Other Mechanical Properties and Thermal Stability of Polymer-Modified Wood

#### 3.4.1. Other Mechanical Properties

**Figure 5:**
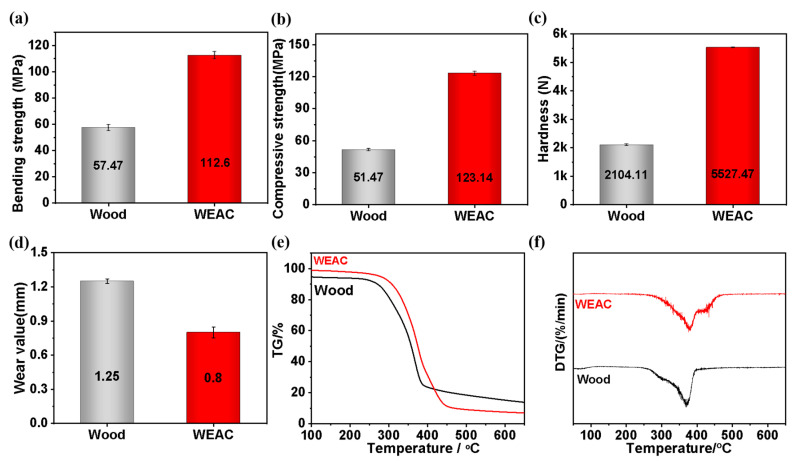
Mechanical properties and heat stability of polymer-modified wood: (**a**) bending strength, (**b**) compressive strength (rift grain), (**c**) hardness, (**d**) wear value, (**e**) TG curves and (**f**) DTG curves.

The bending strength (112.6 MPa), compressive strength (123.14 MPa) and hardness (5527.47 N) of Wood-P (Man-GMA-co-PEGDMA) composites is improved by 101.2%, 139.2% and 162.7%, respectively, compared with unmodified wood, and the wear value is also markedly improved (Figure 5a–d). Their synthetical properties are equivalent to those of high-quality Chinese oak wood [24]. These results fully demonstrate that polymer serves as a reinforcement and plays a positive role in enhancing the mechanical properties of wood.

#### 3.4.2. Thermal Stability

The TG curves shown in Figure 5e uncover that compared with Wood, Wood-P (Man-GMA-co-PEGDMA) composites have a higher initial thermal decomposition temperature [about 275 °C (epitaxial) vs. about 290 °C]. The DTG curves shown in Figure 5f reveal that the maximum thermal degradation rate peak temperature of Wood is around 365 °C, while that of Wood-P (Man-GMA-co-PEGDMA) composites rises to 380 °C, and the second thermal degradation rate peak temperature of Wood-P (Man-GMA-co-PEGDMA) composites is as high as 433 °C. In terms of molecular structure, GMA is polymerized with PEGDMA in the functional monomer system and grafts onto wood with the help of MAN. The synergistic optimization of the three monomers not only improves the structure of the polymer but also enhances the heat stability of wood. Finally, Wood-P (Man-GMA-co-PEGDMA) composites display higher initial thermal decomposition temperature and maximum thermal degradation rate peak temperature than wood.

In brief, the synthetical properties of Wood-P (Man-GMA-co-PEGDMA) composites are obviously improved. This is mainly ascribed to the synergistic optimization of the monomer system in wood, which improved the structure of the polymer and the interfacial interaction between polymer and wood substrate, further enhancing the properties of Wood. This also reflects the rationality of the system constructed.

## 4. Conclusions

In this study, MAN, GMA and PEGDMA were selected to form a target functional monomer system. An organic unsaturated monomer system with low volatility was designed based on the principle of free radical polymerization to construct high-performance modified wood with good interfacial compatibility between polymer and wood, high impact toughness, no formaldehyde release, and compatible mechanical strength, dimensional stability and durability. It was determined that the optimal ratio was nGMA:nPEGDMA = 20:1 (molar ratio), and the optimal MAN content was 6% wt of polymers based on the surface bonding strength of veneered man-made board composites modified by this system. The cell cavity of Wood-P (Man-GMA-co-PEGDMA) composites prepared is fully filled with the polymer in an amorphous state, forming a strong interfacial interaction force between the polymer and the wood substrate, and the resulting epoxy-acrylic polymer displayed an interfacial bonding strength of 1.13 MPa with wood. Additionally, the impact toughness of modified wood was as high as 74.85 KJ/m^2^, which was twice that of wood before modification, and the bending strength, compressive strength, water absorption rate and volume expansion ratio were increased by 96%, 162.7%, 87.8% and 60.7%, respectively. These results indicate that the technical strategy proposed in this study is expected to realize the high-value application of low-quality wood in the field of structural wood. The results of this study render a novel method for building formaldehyde-free, high-performance wood with dimensional stability and toughness.

## Data Availability

The data presented in this study are available upon request from the corresponding author.
